# Quantitative Evaluation of Hemodynamics in the Fontan Circulation: A Cross-Sectional Study Measuring Energy Loss In Vivo

**DOI:** 10.1007/s00246-013-0783-4

**Published:** 2013-09-04

**Authors:** Takashi Honda, Keiichi Itatani, Manabu Takanashi, Eri Mineo, Atsushi Kitagawa, Hisashi Ando, Sumito Kimura, Yayoi Nakahata, Norihiko Oka, Kagami Miyaji, Masahiro Ishii

**Affiliations:** 1 Department of Pediatrics, Kitasato University School of Medicine, Sagamihara, Kanagawa Japan; 2 Department of Cardiovascular Surgery, Kitasato University School of Medicine, 1-15-1 Kitasato, Minami-ku, Sagamihara, Kanagawa 252-0374 Japan; 3 Department of Hemodynamic Analysis, Kitasato University School of Medicine, Sagamihara, Kanagawa Japan

**Keywords:** Energy loss, Fontan circulation, Structural configuration, Diastolic function

## Abstract

Flow energy loss (EL) at the Fontan anastomosis has been thought to reflect flow efficiencies and to influence on hemodynamics in the Fontan circulation and has been often discussed in numerical studies. However, in vivo EL measurements have to date not been reported. We directly measured EL in the Fontan circulation and examined the relationship between the structural configuration and EL, as well as the influence of EL, on the hemodynamics in the Fontan circulation. We performed a catheterization study measuring simultaneous pressure and flow velocity to calculate EL in nine patients (mean age 2.3 ± 0.3 years) 1 year after the Fontan procedure. The measured EL was 9.66 ± 8.50 mW. One patient with left pulmonary artery stenosis recorded the highest EL (17.78 mW), and one patient with bilateral superior vena cava and left phrenic nerve palsy recorded the second highest EL (14.62 mW). EL significantly correlated with time constant tau and weakly with max-d*p*/d*t* during the isovolumic diastolic phase (*r* = 0.795 and −0.574, respectively). EL also correlated with max-d*p*/d*t* during the isovolumic systolic phase (*r* = 0.842) but not with ejection fraction or systemic blood flow (*r* = 0.384 and −0.034, respectively). In conclusion, inefficient structural configuration and phrenic nerve palsy seem to be related with increased in EL at the Fontan anastomosis. Although these preliminary findings also suggest that EL is associated with an impaired relaxation of the ventricle, a long-term study with a large population is warranted to reach such a definitive conclusion.

## Introduction

The Fontan procedure was first reported as a surgical operation for tricuspid atresia in 1971 [6] and is a groundbreaking surgical strategy for single-ventricle patients because it remarkably improves life expectancy and activities of daily living [3, 14]. However, many survivors experience cardiac dysfunction and limited exercise tolerance [7, 8]. Although decreased cardiac function is due to the disadvantage of the Fontan circulation, this mechanism has not been fully elucidated; therefore, it is essential to clarify the hemodynamics in the Fontan circulation.

One of the greatest disadvantages of the Fontan circulation is that it does not have a subpulmonary ventricle to augment the pulmonary circulation. Therefore, the structural configuration of the Fontan anastomosis is believed to easily cause inefficient flow in the pulmonary circulation and has been discussed from the point of view of flow dynamics [2, 4, 5, 12, 15, 18, 19]. Because the flow collision from the superior (SVC) and inferior vena cava (IVC) at the anastomosis causes inefficient flow, there have been several numerical studies with computational simulations to show the hemodynamic details of this flow collision [4, 18]. The evidence derived from computer simulation studies showed several findings regarding the blood flow energy loss (EL) through the total cavopulmonary connection (TCPC). EL is susceptible to types of TCPC procedures [2], pulmonary flow split [18, 19], or pulmonary arterial size [12]. However, although these previous numerical studies have discussed EL, no in vivo clinical measurements of EL have been reported to date, possibly because of the difficulty that measuring EL requires simultaneous pressure and flow velocity data in “all” of the inlets and outlets. Moreover, no previous study has proved whether or not EL significantly influences cardiac performance in Fontan patients.

Therefore, we performed simultaneous measurements of pressure and flow velocity at the SVC and IVC, as well as the bilateral pulmonary artery (PA), and calculated in vivo EL in nine Fontan patients. To our knowledge, this is the first report measuring in vivo EL in the Fontan anastomosis. In the present study, we also measured the parameters expressing cardiac functions of the single ventricle, and examined how they are affected by EL, because the single systemic ventricular function and its effects on exercise tolerances have attracted much attention [1, 9, 17]. The purpose of this study was (1) to evaluate the influence of the structural configuration of the Fontan anastomosis and the complications on EL measured in vivo and (2) to elucidate the influence of EL on systemic single-ventricular functions.

## Materials and Methods

### Patient Characteristics

The present study was a cross-sectional catheterization study measuring pressure and flow velocity simultaneously. Catheter examinations were performed 1 year after the Fontan procedure in nine patients during the period from December 2008 to May 2011 (Table 1). There were two patients with hypoplastic left heart syndrome, two with pulmonary atresia with intact ventricular septum, two with single ventricle (SV), two with double-outlet right ventricle with hypoplastic left ventricle, and one with transposition of the great arteries with a hypoplastic right ventricle. Six patients had a right main ventricle, and the others had a left main ventricle. The patients included five males and four females. Mean age at the time of surgery was 1.2 ± 0.2 years (range 1.0–1.6), and mean age at the time of catheterization was 2.3 ± 0.3 years (range 2.0–2.7). Mean body surface area at the time of catheterization was 0.50 ± 0.04 m^2^ (range 0.44–0.57).

**Table 1:** Patient characteristics

Patient no.	Disease	Age at operation (month)	Age at catheterization (month)	Sex	Main ventricle	Procedure	EL (mW)	Complication	Medication
1	DORV, HLV	16	31	F	R	LT	8.46	None	ACEI, sildenafil
2	SV, SA	15	27	M	L	Intra-atrial conduit	14.62	Bilateral SVC, phrenic nerve palsy	ACEI, diuretic, digitalis
3	DORV, HLV	14	25	M	R	Extracardiac TCPC	5.47	None	None
4	HLHS	19	33	F	R	Extracardiac TCPC	17.78	LPA stenosis	ACEI, diuretic, β-blocker, digitalis
5	SV, SA	12	24	F	R	Extracardiac TCPC	10.48	None	Diuretic, digitalis
6	SV, PA-IVS, TA	13	25	M	L	Extracardiac TCPC	6.32	None	Diuretic
7	TGA(II), HRV	12	24	M	L	Extracardiac TCPC	7.93	None	None
8	PA-IVS, HRV	15	29	F	L	Extracardiac TCPC	10.67	None	ACEI, sildenafil
9	HLHS	15	29	M	R	Extracardiac TCPC	5.26	None	None

*ACEI* angiotensin-converting enzyme inhibitor, *DORV* double-outlet right ventricle, *EL* energy loss, *HLHS* hypoplastic left heart syndrome, *HLV* hypoplastic left ventricle, *HRV* hypoplastic right ventricle, *LPA* left pulmonary artery, *LT* lateral tunnel, *PA-IVS* pulmonary atresia with intact ventricular septum, *SA* single atrium, *SV* single ventricle, *SVC* superior vena cava, *TA* tricuspid valve atresia, *TCPC* total cavopulmonary connection, *TGA* complete transposition of the great arteries

The operations included one lateral tunnel, one intra-atrial conduit, and seven extracardiac TCPCs. The conduit size was 16 mm in all of the patients with intra-atrial conduit or extracardiac TCPCs. No fenestration between the tunnel and the atrium or significant collateral flow was identified. One patient had left phrenic nerve palsy and bilateral SVC, and 1 patient had LPA artery stenosis. Four patients were treated with diuretics, five with a PA dilator, four with an angiotensin-converting enzyme inhibitor, three with digitalis, and one with a β-blocker (Table 1). Written informed consent was obtained from each of the patients’ parents. This study was approved by the Institutional Review Board of Kitasato University Hospital (C09-68).

### Catheterization Method

**Fig. 1:**
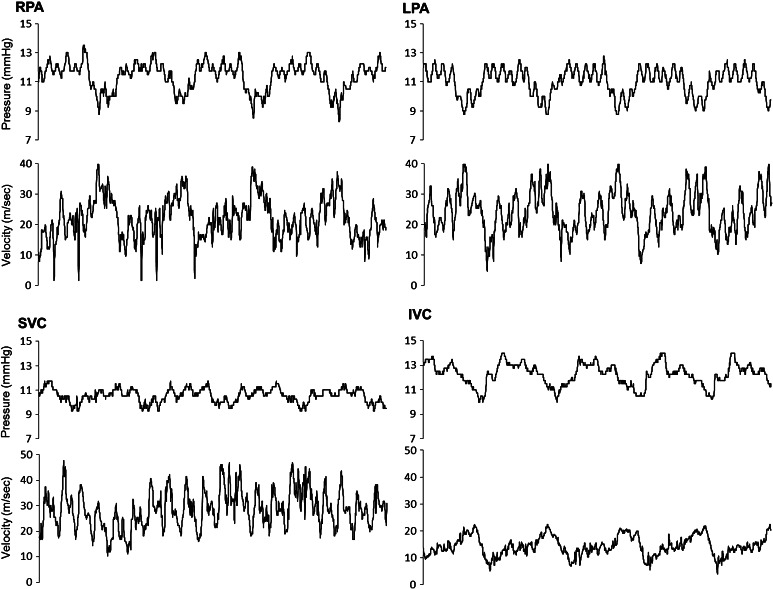
Catheterization method. Intravascular pressure and doppler flow velocity were simultaneously measured at the SVC, the IVC, the RPA, and the LPA

Cardiac catheterizations were performed with the patient under mild systemic anesthesia with ketamine and thiopental, thereby maintaining the patients’ spontaneous breathing. We measured simultaneous intravascular pressure and Doppler flow velocity at the SVC, the IVC, the right pulmonary artery (RPA), and the left pulmonary artery (LPA) using a ComboWire (Volcano Corp., Tokyo, Japan). Data at the RPA and the LPA were taken at the proximal side of the first PA branch. Data at the IVC were taken directly at the cranial side of the marginal section from the hepatic vein. Figure 1 shows an example of simultaneous pressure and velocity data. These data were converted to digital data using the ComboMap Pressure and Flow System (Volcano). We also measured SVC, IVC, RPA, and LPA diameters using cineangiography to assess the cross-sectional vessel lumen area. The pressure of the ventricle was measured, and ventriculography was performed with a contrast agent.

### Calculations

EL was calculated based on the total pressure (TP) defined as:

$${\text{TP}}\; = \;{P}\; + \;\frac{1}{2}{\rho v}^{ 2}$$

$${\text{EL}}\; = \;\sum {{Q}}_{\text{i}} \left( {{P}}_{\text{i}} \;{ + }\;\frac{1}{ 2}{\rho v}_{\text{i}}^{ 2} \right)\;{ - }\;{\sum Q}_{\text{o}} \left( {{P}}_{\text{o}} \;{ + }\;\frac{ 1}{ 2}{\rho v}^{ 2}_{\text{o}} \right)$$

where ρ, Q, P, and v (subscript i indicating the SVC and IVC inlets and subscript _o_ indicating the RPA and LPA outlets) were blood density, volume flow rate, pressure, and velocity, respectively [12, 18, 19]. Pressure and velocity data represented the averaged data of 20 cardiac cycles. Q was calculated as the product of velocity and the cross-sectional area of the vessel lumen. The cross-sectional areas of the bilateral PAs were assumed to be circles; the approximate surface area was calculated using the PA size; the cross-sectional areas of the SVC and IVC were assumed to be elliptical in shape; and the approximate surface area was calculated using the frontal and lateral vessel diameters.

Inlet energy was defined as:

$${\text{Inlet energy}}\; = \;\sum {{Q}}_{\text{i}} \left( {{P}}_{\text{i}} \;{ + }\;\frac{ 1}{ 2}{{\rho v}}^{2}_{\text{i}} \right)$$

To assess the efficiency of the Fontan anastomosis, we calculated EL/inlet energy.All calculations were performed based on the digital converted pressure and velocity data. The calculations were performed using the computer programming language MATLAB (The MathWorks, Natick, MA).

### Ventricular Functions

Ventricular systolic functions were evaluated with a reasonable index of the initial velocity of myocardial contraction (max-dp/dt) during the isovolumic systolic phase (Sdpdt), ejection fraction (EF) in volumetry with ventriculography, and systemic blood flow (Qs), whereas diastolic functions were evaluated using max-dp/dt (Ddpdt), time constant tau in the isovolumic diastolic phase, and end-diastolic pressure (EDP). These data were measured from the ventricular pressure curve obtained by routine cardiac catheterization.

Regarding the basic clinical data, which were based on the catheterization, we also evaluated pulmonary artery wedge pressure (PAWP), central venous pressure (CVP), pulmonary blood flow (Qp), pulmonary vascular resistance (PVR), systemic vascular resistance (SVR), arterial oxygen saturation (Spo_2_), and venous oxygen saturation (Svo_2_). We examined the correlation between these parameters and in vivo EL using simple regression analysis.

## Results

**Fig. 2:**
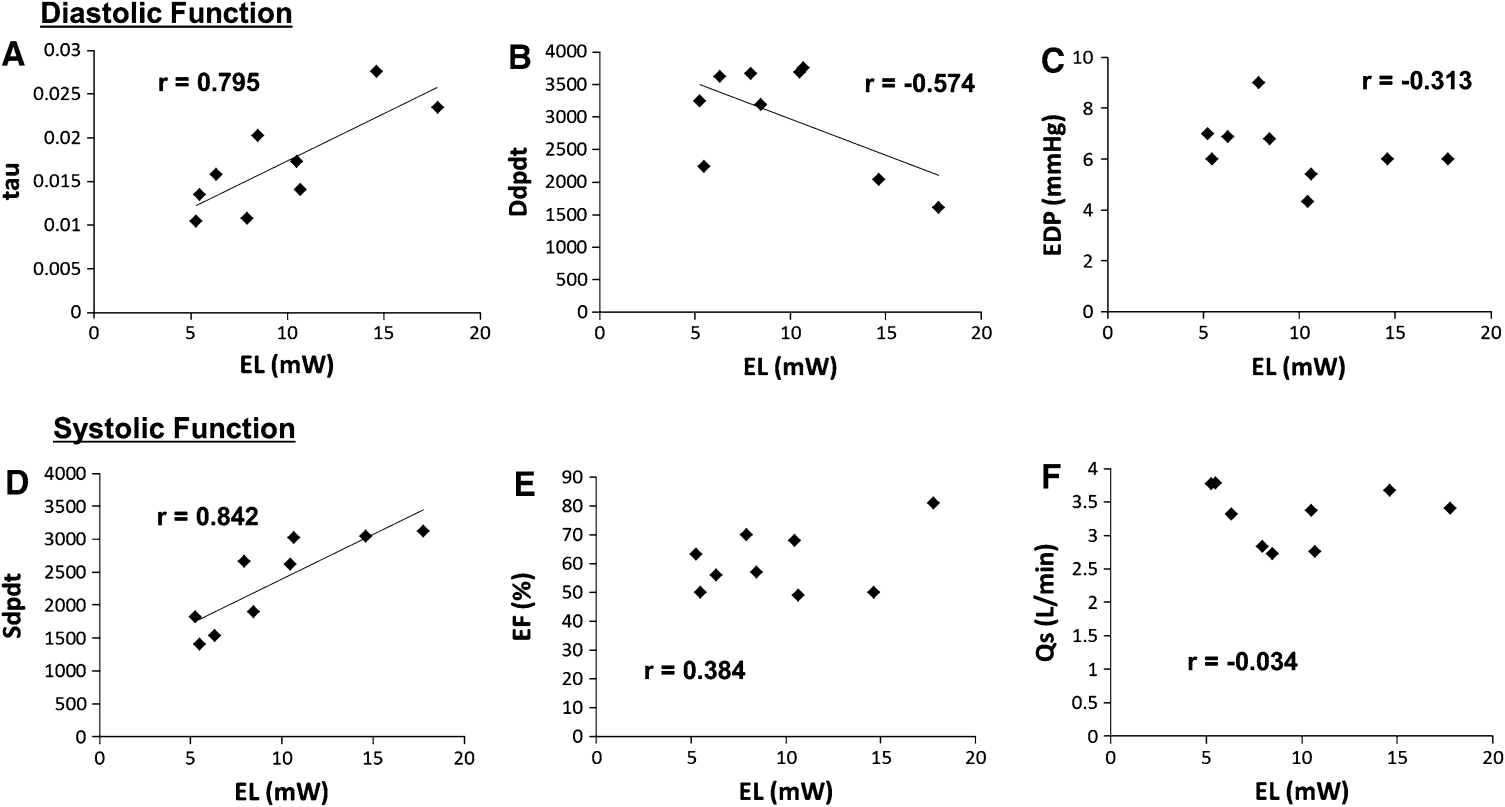
Relationship between ventricular diastolic and systolic function and EL. **a** EL was significantly correlated with time constant tau (*r* = 0.795). **b** EL was weakly correlated with max-dp/dt during Ddpdt (*r* = −0.574). **c** EL was not correlated with EDP (*r* = −0.313). **d** EL was significantly correlated with max-dp/dt during Sdpdt (*r* = 0.842). **e** EL was not correlated with EF (*r* = 0.384). **f** EL was not correlated with Qs (*r* = –0.034)

Direct measured EL was 9.66 ± 8.50 mW (range 5.26–17.78). A patient with phrenic nerve palsy and bilateral SVC recorded the highest EL (17.78 mW) (case no. 4). A patient with LPA stenosis recorded the second highest EL (14.62 mW) (case no. 2). In this patient, the measured pressure gradient in the LPA was 5 mm Hg. The ratio of EL to inlet energy was 17.7 ± 9.4 % (range 6.8–36.6 %). There were no patients with liver dysfunction, protein-losing enteropathy, or thrombosis in the Fontan anastomosis. The clinical information and calculated EL values for each patient are listed in Table 1. Table 2 lists the correlation between EL and catheterization data regarding cardiac function. We also showed the correlation between EL/inlet energy and these data. EL was also significantly correlated with Sdpdt (*r* = 0.842) but not with EF (*r* = 0.384) or Qs (*r* = −0.034) (Fig. 2). EL was also significantly correlated with time constant tau (*r* = 0.795) and weakly with Ddpdt (*r* = −0.574). These results indicate the relationship between high EL and diastolic dysfunction. However, EL had little correlation with EDP (*r* = −0.313). EL and EL/inlet energy did not correlate with any other parameters, such as PAWP, CVP, Qp, PVR, SVR, Sao_2_, or Svo_2_ (Table 2).

**Table 2:** Correlations between EL and cardiac functional parameters

Parameter	EL		EL/inlet energy	
	r	*p*	*r*	*p*
Inlet energy	–0.214	NS	–0.658	NS
Sdpdt	0.842	0.004	0.764	0.016
Ddpdt	–0.574	NS	–0.360	NS
Time constant tau	0.795	0.010	0.868	0.002
EF	0.385	NS	0.0252	NS
EDP	–0.628	NS	–0.398	NS
PAWP	–0.537	NS	–0.240	NS
CVP	–0.004	NS	0.175	NS
Qp	0.070	NS	0.109	NS
Qs	–0.034	NS	–0.060	NS
PVR	0.132	NS	0.028	NS
SVR	–0.190	NS	–0.006	NS
SaO_2_	–0.346	NS	0.135	NS
SvO_2_	–0.005	NS	0.135	NS

*CVP* central venous pressure, *Ddpdt* the max-dp/dt during the isovolumic diastolic phase, *EDP* end diastolic pressure, *EF* ejection fraction, *EL* flow energy loss, *NS* non-significant, *PAWP* pulmonary artery wedge pressure, *PVR* pulmonary vascular resistance, *Qp* pulmonary blood flow, *SaO*_*2*_ hemoglobin oxygen saturation, *Qs* systemic blood flow, *Sdpdt* max dp/dt during the isovolumic systolic phase, *SpO*_*2*_ arterial oxygen saturation, *SvO*_*2*_ venous oxygen saturation, *SVR* systemic vascular resistance

## Discussion

Although EL has been thoroughly discussed and is considered to be an important parameter to evaluate the efficiency of blood flow in relation to the surgical procedures in numerical simulation models of TCPC flow [2, 4, 12, 15, 18, 19], including patient-specific models [2], in vivo measurements in patients’ Fontan circulation have not been reported in the literature to date. Therefore, it remains unsolved whether or not the structural configurations alter EL in the Fontan anastomosis. The effects of EL on the ventricular functions and prognoses of the single-ventricle patients have also been unclear. In the present study, we (1) calculated in vivo EL based on measured simultaneous pressure and flow velocity data, (2) attempted to show the effects of the structural configuration on EL at the TCPC anastomosis, and (3) attempted to elucidate the relationship between EL and the single-ventricular functions.

The characteristics of EL in the TCPC have been mainly examined using computational fluid dynamics studies. A previous computational fluid study reported that EL values ranged from 6.9 to 42.9 mW depending on the type of Fontan procedure used, including LT, intra-atrial conduit, and extracardiac TCPC [2]. Our in vivo EL was 9.66 ± 8.50 mW; therefore, our data were nearly the same compared with EL values reported in previous simulation studies. Compared with previously reported EL values obtained using numerical simulation, EL values obtained in the present study were based on simultaneously measured pressure and velocity data without making any estimations.

In addition, EL could reflect on several physiological phenomena, including pressure relaxation due to pulmonary or cava vessel compliances and respiratory or pulsatile flow fluctuation. Therefore, although EL based on actual data has an inevitable measurement error, we consider that the present study made it possible to approach the true EL values. The present study also showed that the ratio of EL to inlet energy ranged from 6.8 to 36.6 %. We suppose that this difference is not negligible and could affect the hemodynamics of systemic blood flow and/or prognoses of Fontan patients.

In the present study, one patient with LPA stenosis (case no. 4) showed the highest EL value. As we demonstrated in the previous simulation study, we consider that the PA stenosis causes a high-pressure gradient and consequently generates greater EL [12]. In addition, Pekkan et al. [16] reported the possibility that performing virtual angioplasty surgery improves TCPC hemodynamics based on a numeric study. Therefore, it is possible that surgical dilation and plasty for the stenosis may decrease the high pressure gradient, and, subsequently, the EL would likely decrease.

One patient in the present study with bilateral SVC (case no. 2) with predicted flow collision recorded the second highest EL value. Whitehead et al. [19] reported that pulmonary flow splits had a significant effect on EL [18]. We suppose that the bilateral SVC could have disturbed pulmonary flow splits and consequently increased EL. This patient also had left phrenic nerve palsy resulting in inefficient pulmonary flow. In Fontan patients, the effects of respiration on venous flow have been shown to be important because of the dissociation of the ventricular driving forces for pulmonary circulation [10, 11]. Therefore, phrenic nerve palsy could have caused inefficient flow pattern and could have lead to greater EL in the Fontan anastomosis. Concerning the different types of Fontan procedures, Bove et al. [2] reported that EL is decreased in the LT. In the present study, there were no apparent differences in EL between the LT and other procedures; however, a study with a larger population will be needed to verify this.

To evaluate the influence of EL on the systemic hemodynamics, we attempted to elucidate the relationship between in vivo EL and several parameters regarding ventricular function and hemodynamics. As the parameters, we adopted those obtained by catheterization because they are more accurate to reflect ventricular functions and hemodynamics than those obtained by echocardiography, whose echo window is poor, especially in single-ventricle patients. At first, regarding parameters for systolic function, EL was significantly corrected with Sdpdt (*r* = –0.842) but not with relatively global functional parameters, such as EF (*r* = 0.384) or Qs (*r* = –0.034), compared with Sdpdt (Fig. 2). In the present study, it is possible that digitalis was administered to recover the decreased cardiac function in some patients and that it increased Sdpdt in these patients. The three patients treated with digitalis presented with higher Sdpdt levels than those of the other six patients (2928 ± 266 vs 2055 ± 645 mmHg/s, respectively). In addition, in these three patients, the EL values were greater than those in the other six patients (14.3 ± 3.7 vs 7.4 ± 2.0 mW, respectively). Therefore, we cannot conclude that EL directly reflected the increased systolic function. To more objectively and accurately analyze the relationship between EL and systolic function, we must also evaluate the effect of digitalis in a future study with a larger population.

Regarding parameters representing diastolic function, EL also significantly correlated with time constant tau (*r* = 0.795) and weakly with Ddpdt (*r* = –0.574) (Fig. 2). However, EL had little correlation with EDP (*r* = −0.313). Ddpdt and time constant tau are parameters that reflect the relaxation faculty, whereas EDP reflects ventricular stiffness; therefore, these results indicated the possibility that there were some relationships between high EL in the Fontan anastomosis and the impaired relaxation faculty of the SV. In patients after Norwood procedures, it was reported that EL could be workload for the main ventricle [13]. In the Fontan circulation, it is possible that EL itself can be an afterload in a serial circulation and can cause workload for an SV.

Previous studies of single-ventricular functions compared with biventricular hearts [1, 17] showed that Fontan patients had almost equal baseline contractility and had the same ventricular stiffness but decreased relaxation compared with normal controls [17]; however, the mechanism underlying these relationships remains unclear. The preliminary data supported the possibility that high EL would be one of the factors that impairs relaxation of the ventricle. However, cardiac function in the Fontan circulation can be affected by many other factors, such as underlying disease, ventricular dominance, medication therapy, characters of myocardium, types of TCPC, and surgical strategy of procedures undergone before the Fontan procedure. The present study is heterogenous in these characteristics, and these differences could influence the parameters regarding cardiac functions, although the timing of operation and catheterization are homogenous. Therefore, a study with a larger population is warranted to definitively prove this conclusion. In addition, medium and long-term studies are also needed because high EL would induce a continuous increased workload and have a long-term effect.

### Limitations

One of the limitations of the present study is that the effects of the body position and muscular pump could not be determined because the measurement was performed with the patient under sedation. Marsden et al. [15] proposed that exercise should be taken into consideration to provide more realistic evaluations of TCPC performance based on their simulation study. Other approaches should be adopted to evaluate the in vivo muscular pump effects on the Fontan circulation. Another limitation is the measurement error, especially of the velocity. A single point was used for vessel velocity and for calculating the flow amount, even though the point of the catheter was confirmed at the center using cineangiography and velocity distributions inside the vessels were taken into account. Another limitation is that the number of patients is small, especially when we statistically analyze the data. In addition, although high EL levels were recorded in one patient with LPA stenosis and in one patient with bilateral SVC and phrenic nerve palsy, a study with a larger population, including patients with these complications, is needed to verify the influence of these structural configurations and complications on EL. However, this method measuring in vivo EL enabled us to overcome many limitations associated with the previous simulation study.

## Conclusion

To our knowledge, this is the first attempt to measure EL in vivo in the Fontan anastomosis. This study showed that an inefficient structural configuration of the Fontan anastomosis can cause greater EL and that a surgical scheme to minimize EL would result in better prognoses for single-ventricle patients. These preliminary data also indicated an association between EL and impaired relaxation of the ventricle. However, this was a small-cohort study; therefore, a future long-term study with a larger population is warranted to confirm this association.
